# Metal-on-Metal Hip Prostheses and Systemic Health: A Cross-Sectional Association Study 8 Years after Implantation

**DOI:** 10.1371/journal.pone.0066186

**Published:** 2013-06-10

**Authors:** Jennifer R. Prentice, Matthew J. Clark, Nigel Hoggard, Allison C. Morton, Claire Tooth, Martyn N. Paley, Ian Stockley, Marios Hadjivassiliou, J. Mark Wilkinson

**Affiliations:** 1 Department of Human Metabolism, University of Sheffield, Sheffield, United Kingdom; 2 Department of Cardiology, Sheffield Teaching Hospitals NHS Foundation Trust, Sheffield, United Kingdom; 3 Department of Neurology, Sheffield Teaching Hospitals NHS Foundation Trust, Sheffield, United Kingdom; 4 Department of Orthopaedics, Sheffield Teaching Hospitals NHS Foundation Trust, Sheffield, United Kingdom; University of Michigan, United States of America

## Abstract

There is public concern over the long term systemic health effects of metal released from hip replacement prostheses that use large-diameter metal-on-metal bearings. However, to date there has been no systematic study to determine which organs may be at risk, or the magnitude of any effect. We undertook a detailed cross-sectional health screen at a mean of 8 years after surgery in 35 asymptomatic patients who had previously received a metal-on-metal hip resurfacing (MoMHR) versus 35 individually age and sex matched asymptomatic patients who had received a conventional hip replacement. Total body bone mineral density was 5% higher (mean difference 0.05 g/cm^2^, P = 0.02) and bone turnover was 14% lower (TRAP 5b, mean difference −0.56IU/L, P = 0.006; osteocalcin, mean difference −3.08 ng/mL, P = 0.03) in the hip resurfacing versus conventional hip replacement group. Cardiac ejection fraction was 7% lower (mean absolute difference −5%, P = 0.04) and left ventricular end-diastolic diameter was 6% larger (mean difference 2.7 mm, P = 0.007) in the hip resurfacing group versus those patients who received a conventional hip replacement. The urinary fractional excretion of metal was low (cobalt 5%, chromium 1.5%) in patients with MoMHR, but creatinine clearance was normal. Diuretic prescription was associated with a 40% increase in the fractional excretion of chromium (mean difference 0.5%, P = 0.03). There was no evidence of difference in neuropsychological, renal tubular, hepatic or endocrine function between groups (P>0.05). Our findings of differences in bone and cardiac function between patient groups suggest that chronic exposure to low elevated metal concentrations in patients with well-functioning MoMHR prostheses may have systemic effects. Long-term epidemiological studies in patients with well-functioning metal on metal hip prostheses should include musculoskeletal and cardiac endpoints to quantitate the risk of clinical disease.

## Introduction

There is public concern about the potential systemic health effects of metal exposure in patients who have received large diameter (≥36 mm) metal-on-metal hip prostheses [1], however there is little data available to quantitate which systems may be affected or the magnitude of any effect [2]. The Food and Drug Administration (FDA) in the United States has recently (May 6^th^, 2012) instructed manufacturers of large diameter metal-on-metal hip prostheses to conduct cross-sectional studies covering the period from implantation out to 8 years after surgery in order to quantitate the adverse local and systemic effects of metal exposure from these devices (http://www.fda.gov/MedicalDevices/Safety/AlertsandNotices/ucm335775.htm, accessed May 13^th^, 2013). The FDA has also advised physicians that asymptomatic patients at risk of increased metal release and symptomatic patients should be clinically monitored for cardiovascular, neurological, renal, and thyroid signs and symptoms (http://www.fda.gov/MedicalDevices/ProductsandMedicalProcedures/ImplantsandProsthetics/MetalonMetalHipImplants/ucm241667.htm, accessed May13th, 2013). Cobalt and chromium are the principal metals released by metal-on-metal hip prostheses [3], [4], including metal-on-metal hip resurfacing (MoMHR). The evidence base for the FDA recommendations in relation to systemic disease derives from case reports of grossly elevated metal levels associated with mal-functioning prostheses, or is translated from their toxicology in animal studies, and accidental or occupational over-exposure in humans [5], [6], [7], [8], [9].

It is estimated that world-wide approximately 1 million patients have received a hip replacement that uses a large-diameter metal-on-metal bearing, and the majority of these patients have well-functioning devices [10]. Well-functioning prostheses also release metal species into the systemic circulation over a prolonged period after surgery. Steady state median blood cobalt and chromium concentrations over 10 years in patients with well-functioning devices are between 1.5 and 2.3 µg/L, and are 10-fold higher than normal physiological concentrations [11], [12]. The systemic effects of this prolonged exposure to low elevated metal levels is unknown and, to date, unstudied [2]. We have recently shown that concentrations of cobalt and chromium equivalent to blood levels after MoMHR affect human bone cell viability and function *in-vitro* [13]. Further, Linna et al [14], found cobalt workers exposed to a blood cobalt level of 2.5 µg/L over 9 years had echocardiographic evidence of altered left ventricular function versus unexposed controls.

Direct evidence for effects of chronic exposure to metal on systemic organ function in patients with well-functioning implants is needed to inform appropriate endpoints for the studies now required of manufacturers, and to inform the appropriate clinical monitoring of patients. We conducted a cross-sectional, in-depth, systematic health screen in patients with well-functioning MoMHR 8 years after implantation and an individually-matched group of patients who received a conventional total hip arthroplasty (THA) in order to determine which organ systems may be susceptible to altered function following insertion of these implants.

## Patients and Methods

### Ethics, design, setting, and participants

We undertook this single-centre cross-sectional study at a teaching hospital in Sheffield, United Kingdom. Patients were recruited from the community between November 24^th^, 2009 and May 20^th^, 2010. The study was approved by South Yorkshire Research Ethics Committee, and all patients provided written informed consent prior to participation. The study design was reviewed by a statistician at Sheffield Clinical Trials Research Unit, and its conduct and dissemination of results were reviewed by Sheffield Lay Advisory Panel for Bone Research.

The MoMHR exposure patients comprised individuals who had undergone surgery for osteoarthritis not less than 5 years previously. The non-exposure group comprised individually-matched osteoarthritis patients who had received a conventional THA using a non-metal-on-metal bearing. All patients were recruited from the operating records of a single surgeon. The MoMHR participants were identified first and individually-matched THA participants were then identified from the same surgeons operating records using the following matching criteria: Age (within 3 years), gender, and time since hip surgery (within 2 years). The exclusion criteria were any revision surgery for complications of hip arthroplasty, known inflammatory arthropathy or metabolic bone disease, use of pharmacological doses of estrogen, progestin, androgen, calcitonin, glucocorticoids, or dietary supplements of calcium or vitamin D within the previous 12 months, any previous use of bisphosphonate or fluoride therapy, pregnancy, and those patients in whom magnetic resonance imaging (MRI) was contra-indicated.

Blood, plasma, and urinary cobalt and chromium were measured by inductively-coupled plasma-mass spectroscopy (ICP-MS). Blood samples were collected via plastic cannula using the last draw method directly into trace element collection tubes. Metal assays were performed by two independent laboratories that participate in National trace element measurement quality assurance schemes. The mean value of the two laboratories was used for analyses to reduce bias arising from inter-laboratory measurement variability. Serum protein and iron status were also quantitated to identify potential bias between study groups due to trace metal bioavailability.

### Outcomes

Total body bone mineral density (TB-BMD) and body composition were measured by dual energy x-ray absorptiometry using an Hologic Acclaim fan-beam densitometer (Hologic Inc, Bedford, MA) with manual exclusion of regions containing metallic prostheses from the analysis. Biochemical markers of bone turnover were measured by automated electro-chemiluminescent assay or by manual enzyme-linked immunosorbent assay in fasting morning serum or 24-hour urine samples, and according to manufacturer’s instructions. Cardiac function was assessed using the New York Heart Association (NYHA) functional classification for cardiac failure and by trans-thoracic echocardiography in accordance with guidelines set by the British Society of Echocardiographers. All examinations were made using the same GE Vivid 7 ultrasound machine (GE Healthcare, Freiberg, Germany). Twelve-lead electrocardiography was used to identify conduction defects using a MAC 5500 resting ECG analysis system (GE Healthcare).

Pre-morbid IQ was measured using the Wechsler test of adult reading (WTAR) and the Hospital Anxiety and Depression scale was administrated to test for any significant differences in these influential variables between the groups. A battery of standardized neuropsychological tests was performed, including: verbal learning by California verbal learning test (CVLT-II); construction skills and recall of visual information using the adult memory and information processing battery (AMIPB) complex figure; spatial perception using the line orientation test from the repeatable battery for the assessment of neuropsychological status (RBANS); attention and working memory using the digit-span subtest of the Wechsler Adult Intelligence Scale (WAIS-III); psychomotor speed using the digit symbol coding and digit symbol search subtests of WAIS-III; verbal fluency using timed word naming for both phonemic and semantic domains.

Renal function was measured by serum and urinary chemistry, and creatinine clearance was calculated using the Mosteller formula [15]. Renal injury was quantitated by manual immuno-assay of urinary renal tubular enzymes previously shown to be elevated in chronic kidney injury [16]. Urinary KIM-1, NAG, and NGAL were measured from 24-hour urine collection by quantitative sandwich enzyme immunoassay (NGAL and KIM-1, R+D Systems, Minneapolis, MN, USA; NAG, USCN Life Sciences Inc., Wuhan, China). Urinary fractional excretion of metal relative to creatinine was also calculated. Hepatic function, injury, and metal accumulation were assessed by liver function tests, coagulation screen, fasting lipid profile, and by gradient echo T2* weighted MRI with varying echo time of the liver and spleen, respectively. All MRI examinations were made using the same 3.0 Tesla Philips MRI scanner (Philips Intera, Best, Netherlands). Endocrine function was evaluated by serum assay of hypothalamo-pituitary axis hormones and pituitary volume was measured by MRI. Insulin resistance was measured by serum assay of fasting glucose and insulin and expressed as the glucose to insulin ratio [17].

### Statistical analysis

The study was powered for the primary outcome measure of TB-BMD. For a population mean TB-BMD of 0.80 g/cm^2^ (standard deviation 0.06 g/cm^2^) a sample size of 35 patients per group had 80% power to detect a 5% difference in TB-BMD between patient groups, assuming a within-pair correlation of 0.6 and using a 2-tailed paired t-test with an alpha of 0.05. All between-group analyses of continuous data were conducted using the paired t-test, or the Wilcoxon test, where appropriate, and categorical data were analysed using the chi-squared test.

## Results

### Participants and metal levels

**Figure 1:**
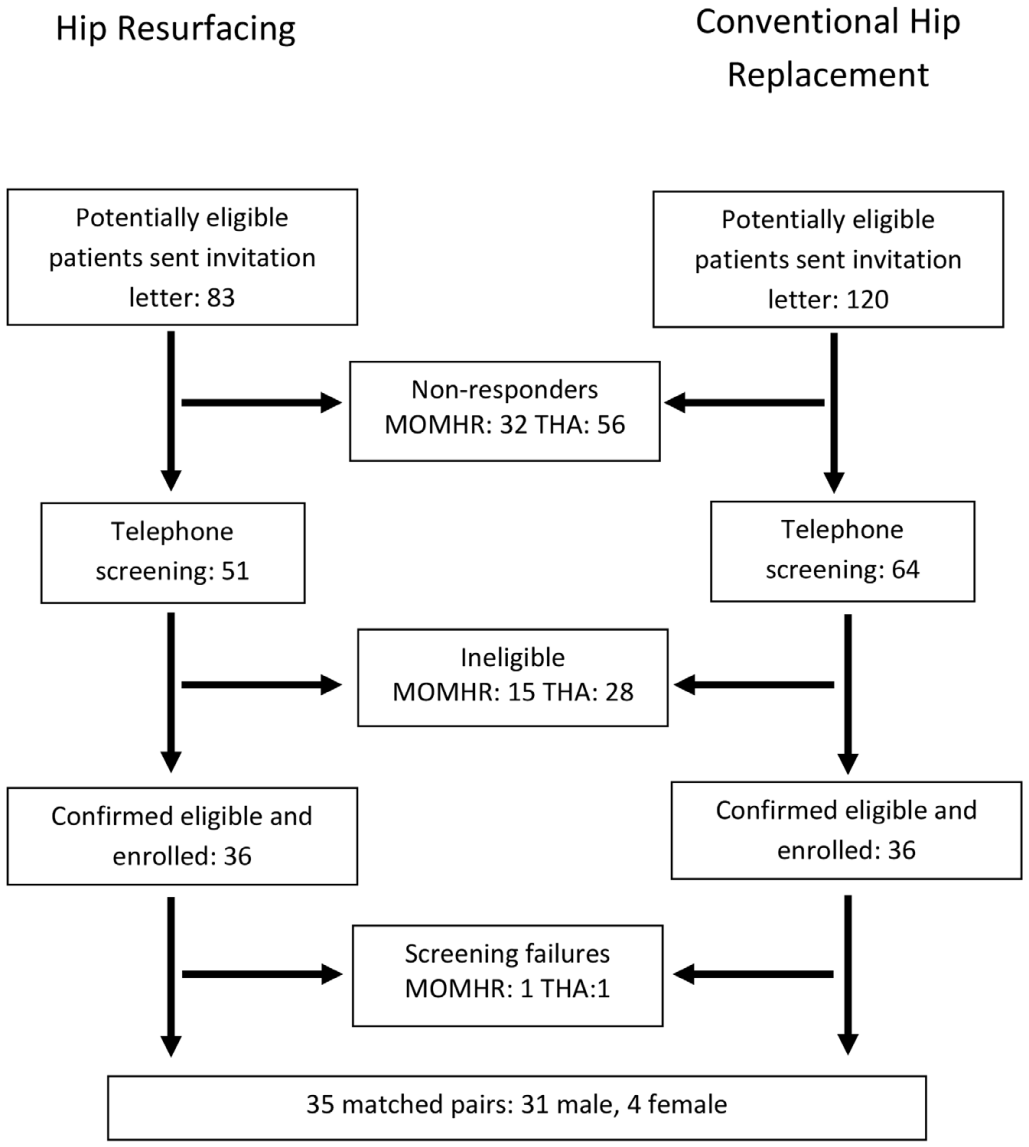
Recruitment flow chart. MoMHR  =  metal-on-metal hip resurfacing, THA  =  conventional hip replacement using a non-metal-on-metal bearing.

Between November 2009 and May 2010, 203 (83 with MoMHR and 120 with THA) potentially eligible patients were invited to participate (Figure 1). We received no response or a decline from 32 of the MOMHR patients and 56 of the THA patients. In the MoMHR group a further 15 patients were excluded for the following reasons: contra-indication to MRI (n = 3), inflammatory arthropathy or metabolic bone disease (n = 3), recent arthroplasty to other joints (n = 3), MoMHR revision (n = 1), use of calcium dietary supplements (n = 2), and current illness (n = 3). In the THA group 28 patients were excluded for the following reasons: complications or revision of the prosthesis (n = 10), failure of the matching criteria (n = 7), inflammatory arthropathy or metabolic bone disease (n = 3), glucocorticoid treatment (n = 3), contra-indication to MRI (n = 3), recent arthroplasty to other joints (n = 1) and subject withdrawal (n = 1). One further patient from each group was excluded after enrolment for screening failure (1 for cancer and the other for an intra-orbital foreign body that precluded MRI investigation). The age distribution of those patients invited to participate but were not enrolled was similar to those who participated (age 60±1years versus 59±1 in recruits, P = 0.53), although a greater proportion were female (M:F  =  98:33 versus 62:8 in recruits; P = 0.02).

Thirty-five pairs of age, gender and time since surgery matched patients completed the study (Table 1). The patients in both groups were also well-matched for body composition, general health measured by EQ-5D and EQ-VAS, Oxford hip score, and pre-arthroplasty risk of osteoporotic fracture by FRAX score. In the MoMHR group 32 patients had received the Birmingham hip resurfacing prosthesis (Smith & Nephew Inc., Andover, MA; FDA approved 2006) and 3 the ASR prosthesis (DePuy Inc., Warsaw, IN; not FDA approved). In the THA group 17 patients received a metal-on-polyethylene bearing, 17 received a ceramic-on-ceramic bearing, and in one a ceramic-on-polyethylene bearing. Median blood, plasma, and urinary cobalt and chromium were 5 to 50 fold higher in MoMHR versus THA patients (P<0.0001). However, blood median and interquartile range metal levels were below guidance threshold concentration of >7 µg/L suggestive of prosthesis mal-function, and set by the Medicines and Healthcare Products Regulatory Agency (MHRA) in the United Kingdom (update MDA/2012/036; 25 June 2012). In the THA group blood, plasma and urinary cobalt concentrations were below the ICP-MS detection limit of 0.3 µg/L in 6, 24, and 27 of the 35 patients, respectively; and chromium concentrations were below this limit in 21, 12, and 22 patients, respectively.

**Table 1:** Patient characteristics and metal levels.

Characteristic	MoMHR (n = 35)	THA (n = 35)
Age at surgery (years)	51.2±6.6	51.8±8.2
Gender (M:F)	31:4	31:4
Time since surgery (years)	8.1±1.8	7.8±2.4
Bilateral: Unilateral hip replacement	11:24	12:23
Bearing diameter (mm)	50 (46 to 52)	28 (22 to 28)
Height (m)	1.74 (0.07)	1.72 (0.08)
Weight (Kg)	86.5 (17.4)	84.9 (15.9)
Body mass index (Kgm^−2^)	28.6 (4.7)	28.4 (3.6)
Fat mass (Kg)	21.2 (9.1)	22.2 (6.9)
Lean mass (kg)	60.9±9.9	58.4±11.1
Body fat (%)	25.1±6.3	27.3±5.6
Body surface area (m^2^)	2.04 (0.23)	2.01 (0.23)
Oxford hip score	46 (43 to 48)	46 (40 to 48)
EQ-5D	1.0 (0.7 to 1.0)	0.9 (0.7 to 1.0)
EQ-VAS	84.7 (11.2)	81.5 (16.7)
‘FRAX’ 10-year major osteoporotic fracture risk (%)	4.7 (2.1)	4.2 (1.5)
*Whole blood cobalt (µg/L)	1.75 (1.11 to 6.11)	0.38 (0.33 to 0.55)
*Whole blood chromium (µg/L)	1.27 (1.04 to 3.91)	<0.30 (<0.30 to <0.30)
*Plasma cobalt (µg/L)	1.48 (0.90 to 5.62)	<0.30 (<0.30 to <0.30)
*Plasma chromium (µg/L)	2.51 (1.61 to 7.07)	<0.30 (<0.30 to 0.31)
*Urinary cobalt (µg/L)	7.47 (4.13 to 17.47)	<0.30 (<0.30 to <0.30)
*Urinary chromium (µg/L)	3.12 (1.54 to 7.23)	<0.30 (<0.30 to <0.30)

Normally distributed data are presented as mean ±SD, and non-normally distributed data as median (IQR). Analysis is MoMHR versus THA. Continuous data were analyzed by paired t-test or Wilcoxon test; categorical data were analyzed by either Chi-squared test. *P<0.0001, P>0.05 for all other comparisons.

### Outcomes

**Figure 2:**
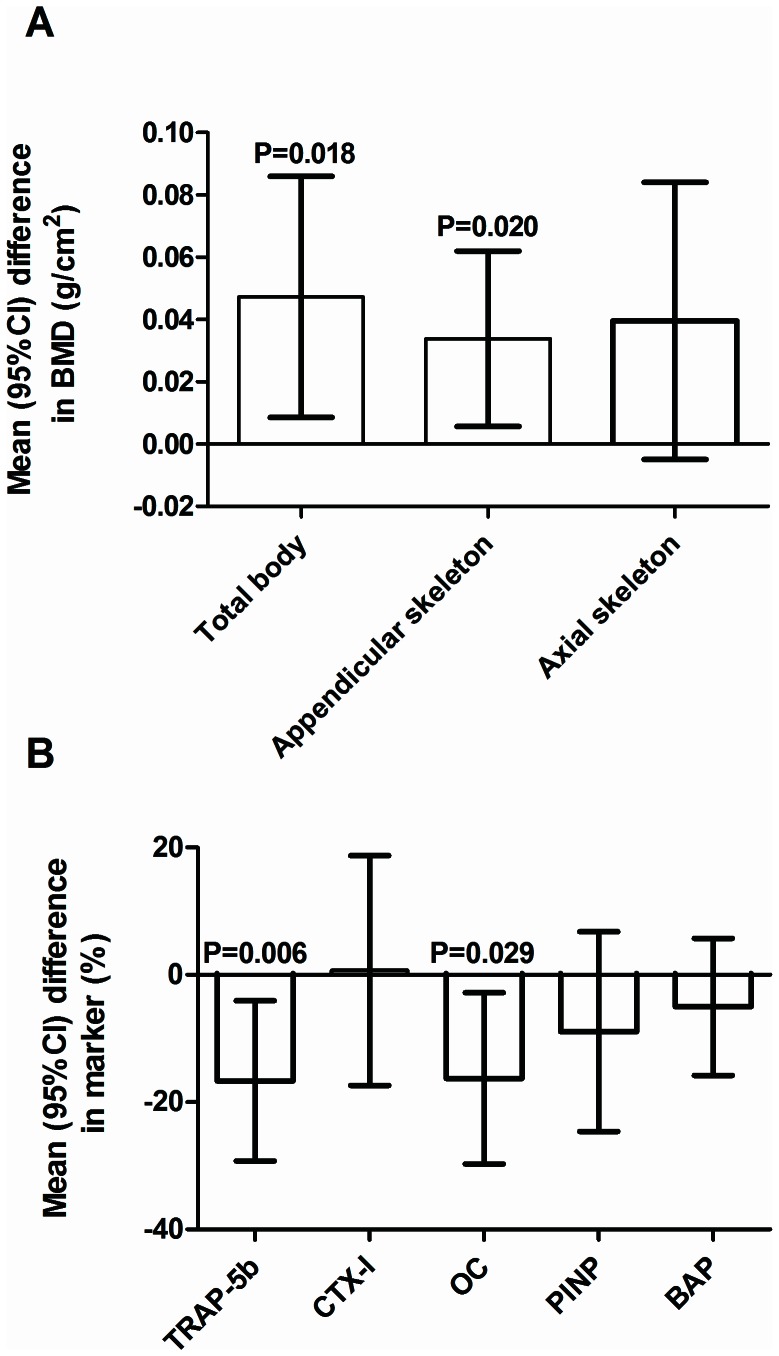
Bone endpoints in MoMHR versus THA patients. A) Mean difference in bone mineral density at various body sites, and B) differences in bone turnover markers between the patient groups. TRAP-5b = Tartrate-resistant acid phosphatase 5b, CTX-I  =  C-telopeptide of type I collagen, OC =  osteocalcin, PINP =  N-terminal propeptide of type-I procollagen, BAP =  bone-specific alkaline phosphatase. Comparison is the difference in endpoint in MoMHR versus THA patients by paired t-test.

TB-BMD was 5% higher in the MoMHR versus the THA group (Figure 2a: 1.04 g/cm^2^ versus 1.00 g/cm^2^, mean difference 0.05 g/cm^2^; 95% confidence interval 0.01 to 0.09: P = 0.02). Within the appendicular skeleton (upper limb), BMD was 4% higher in the MoMHR versus the THA group (0.86 g/cm^2^ versus 0.83 g/cm^2^, mean difference 0.03 g/cm^2^; 0.01 to 0.06: P = 0.02). Within the axial skeleton (spine and ribs), BMD was 5% higher in the MoMHR versus THA group (0.83 versus 0.79, mean difference 0.04 g/cm^2^; 0.00 to 0.08: P = 0.08). Bone formation, measured by serum osteocalcin was 14% lower (mean difference −3.08 ng/mL; −5.83 to −0.33: P = 0.03) and osteoclast number measured by serum tartrate-resistant acid phosphatase 5b was 14% lower (mean difference −0.56IU/L; −0.96 to −0.17: P = 0.006) in the MoMHR patients versus the THA group (Figure 2b).

Patients in both groups had similar levels of risk factors for heart disease, treatment, cardiac symptoms by NYHA score, resting heart rate, and evidence of conduction abnormalities by ECG (Table 2, P>0.05, all comparisons). Trans-thoracic echocardiography demonstrated that cardiac ejection fraction was 7% lower (Table 2; 60% versus 65%; mean absolute difference −5%; 95% CI −9.4 to −0.19: P = 0·04) and left ventricular end-diastolic diameter was 6% larger (48.9 mm versus 46.2 mm, mean difference 2.7 mm; 0.80 to 4.57: P = 0.007) in the MoMHR versus the THA patients.

**Table 2:** Cardiac endpoints.

Characteristic	MoMHR (n = 35)	THA (n = 35)
Alcohol (units/week)	14.6 (15.9)	17.1 (14.7)
Smoking: (never/current/Ex.>5years/Ex.<5 years)	22/4/2/7	23/3/1/8
Treated or known hypertension	6	11
History of diabetes mellitus	3	3
Cholesterol (mmol/L)	5.1±1.1	5.3±1.3
Triglyceride (mmol/L)	1.3±0.8	1.4±0.9
High density lipoprotein cholesterol (mmol/L)	1.2±0.4	1.2±0.4
Low density lipoprotein cholesterol (mmol/L)	3.3±0.9	3.4±1.1
Total/high density lipoprotein cholesterol ratio	4.4±1.4	4.6±1.7
Statin treatment	7	12
History of ischaemic heart disease	2	0
NYHA cardiac failure score	1 (1 to 1)	1 (1 to 1)
Conduction defect identified by ECG	3	2
Resting heart rate	63±12	62±9
Left ventricular end-diastolic diameter (mm)**	49±4	46±4
End systolic diameter (mm)	31±6	29±4
Iinterventricular septum thickness(mm)	11±2	11±2
Posterior wall thickness (mm)	11±1	10±2
Ejection fraction (%)*	60±9	65±7

Normally distributed data are presented as mean ±SD, and non-normally distributed data as median (IQR). Analysis is MoMHR versus THA. Continuous data were analyzed by paired t-test or Wilcoxon test; categorical data were analyzed by either Chi-squared test. *P<0.05, **P<0.01, P>0.05 for all other comparisons.

**Figure 3:**
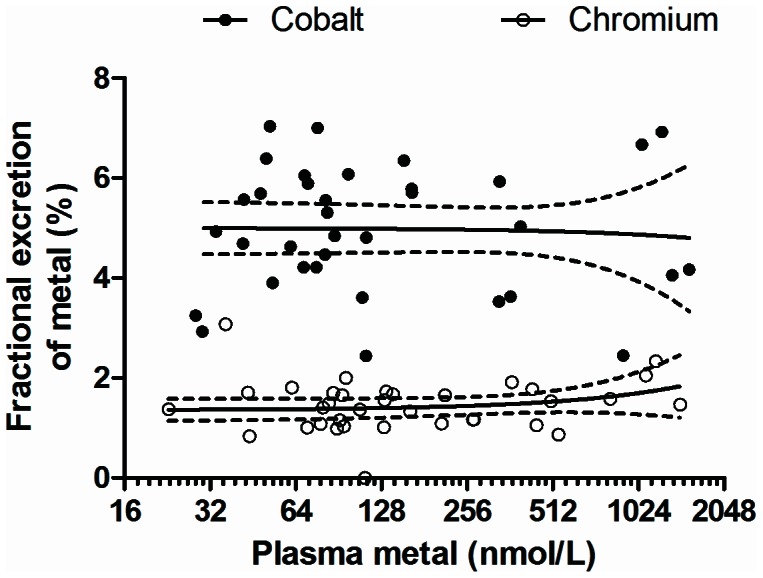
Urinary fractional excretion of cobalt and chromium versus plasma metal level in MoMHR patients. Line represents regression slope and dotted line represents 95% confidence interval. Comparison is fractional excretion of cobalt versus fractional excretion of chromium by linear regression analysis (P<0.0001).

Patients in the MoMHR group had similar levels of neuropsychological function measured using standardized tests of cognitive, behavioural, and psychological function versus patients in the THA group (P>0.05, Table 3). Hospital anxiety and depression scores were also similar between the groups (P>0.05). Serum electrolytes, creatinine clearance, and enzymatic markers of renal tubular damage were similar between groups (Table 4; P>0.05, all comparisons). In the MoMHR group daily urinary excretion of cobalt versus chromium was 12.2 µg (interquartile range 8.6 to 45.6) versus 6·2 µg (4.0 to 18.5, P<0.0001). The fractional excretion of cobalt (relative to creatinine) versus chromium was 5% versus 1% (Figure 3, P<0.0001). The fractional excretion of both metals was constant and independent of plasma metal concentration (gradient of slope  =  zero, P>0.05). Five of the 35 subjects in the MoMHR group were prescribed diuretics. Urinary fractional excretion of chromium in this sub-group was 40% higher than in the non-users (mean difference 0.5%, 0.1 to 1.0; P = 0.03). Urinary metal levels were undetectable in the majority of patients in the THA group, precluding estimation of daily and fractional metal excretion.

**Table 3:** Neuropsychological and psychological endpoints.

Test variable	MoMHR (n = 35)	THA (n = 35)
Construction skills by copy (%)	97±5	97±3
Immediate recall of visual information (%)	74±16	69±15
Delayed recall of visual information (%)	73±15	68±16
Retained recall of visual information (%)	100±12	99±9
Spatial perception by Line Orientation (number correct)	19±2	18±2
Verbal Learning (total correct over trials 1 to 5)	62±12	64±11
Psychomotor speed (sum of scaled scores)	20±4	21±5
Attention and working memory (number of forward digits correct)	7±1	7±1
Attention and working memory (number of backward digits correct)	5±2	5±1
Attention and working memory (difference between forwards and backwards span)	2±1	2±1
Verbal fluency- semantic (number of animals listed in 60 seconds)	19±4	20±4
Verbal fluency- phonetic (sum of number of words listed beginning with each of F, A and S in 60 seconds)	40±13	41±11
Pre-morbid Intelligence Quotient (full scale scored IQ calculated using United Kingdom normative data)	99±10	101±9
Hospital anxiety score	4 (3 to 6)	4 (2 to 8)
Hospital depression score	3 (2 to 6)	2 (1 to 6)

All test results were adjusted for by pre-morbid Intelligence Quotient using the Wechsler test for adult reading (WTAR). Values are mean±standard deviation or median (interquartile range). Analysis is MoMHR versus THA by paired t-test or Wilcoxon test; P>0.05, all comparisons.

**Table 4:** Serum markers of renal, hepatic, and endocrine function and injury.

Serum analyte	MoMHR (n = 35)	THA (n = 35)
*Renal function and injury*		
Sodium (mmol/L)	144.5±2.7	144.3±2.8
Potassium (mmol/L)	4.5 ±0.4	4.5±0.3
Urea (mmol/L)	5.6±1.9	5.1±1.1
Creatinine (mmol/L)	91.2±20.0	88.6±18.3
Creatinine clearance (mL/min/1.73 m^2^)	111.6±35.3	99.1±28.0
^‡^Kidney injury molecule-1 (ng/mL)	0.7±0.5	0.8±0.1
^‡^N-acetyl-β-(D)-glucosamidase (ng/mL)	25.2±13.4	26.3±16.7
^‡^Neutrophil gelatinase-associated lipocalin (ng/mL)	2.9 (1.8 to 4.7)	2.9 (1.8 to 3.8)
***Hepatic function and injury***		
Total protein (g/L)	71.7±3.8	73.5±4.8
Albumin (g/L)	44.6±2.3	45.1±3.3
Globulin (g/L)	27.1±3.7	28.4±3.0
Serum ferritin (µg/L)	174.1±134.0	193.1±137.4
Serum transferrin receptor	2.6±0.4	2.6±0.4
Adjusted calcium (mmol/L)	2.3±0.1	2.4±0.1
Total bilirubin (µmol/L)	13.4 ±7.8	12.4±6.1
Alkaline phosphatase (IU/L)	61.7±15.3	63.3±15.6
Alanine transaminase (IU/L)	31.7±9.6	29.7±10.4
Prothrombin time (seconds)	10.6±0.7	10.6±0.4
Activated partial thromboplastin time (seconds)	30.9±3.0	31.7±3.7
Fibrinogen (g/L)	3.3±0.8	3.4±0.7
***Endocrine function***		
Thyroid stimulating hormone (mIU/L)	2.3 ±1.4	2.2±1.2
Free tri-iodothyronine (pmol/L)	5.4±0.6	5.4±0.6
Luteinising hormione (IU/L)	4.8 (3.5 to 12.4)	4.8 (3.5 to 8.8)
Follicle stimulating hormone (IU/L)	6.1 (4.6 to 21.8)	6.4 (4.4 to 12.4)
Prolactin (mIU/L)	145.5±64.3	153.2±93.0
Cortisol (nmol/L)	423.7±136.7	397.9±107.7
^‡^Testosterone (nmol/L)	15.3±6.3	14.2±5.7
Oestradiol (mIU/L)	35.4±9.5	31.5±12.9
Growth hormone (µg/L)	0.4 (0.1 to 0.8)	0.40 (0.1 to 1.0)
Fasting insulin (mIU/L)	11.1±6.3	10.2±6.0
Fasting glucose (mmol/L)	6.2±1.0	5.9±1.1
Fasting glucose to insulin ratio (mmol/mIU)	0.8±0.5	0.9±0.9

Values are mean ± standard deviation or median (interquartile range). Analysis is MoMHR versus THA by paired-t test or Wilcoxon test. P>0.05 all comparisons. ^‡^Marker assayed in men only (n = 31 per group).

Serum markers of liver synthetic function and injury, fasting lipids, and clotting factors were similar between groups (Table 4; P>0.05). Serum protein, albumin, ferritin and transferrin receptor concentrations, which may affect biological availability of metal, were also similar between groups (P>0.05). Patients in the MoMHR group had increased magnetic resonance T2* relaxation-time for the liver (mean difference 0.94msec; 0.09 to 1.79: P = 0.03) and spleen (mean difference 0.87msec; 0.05 to 1.68: P = 0.04) versus THA patients, consistent with metal deposition within these organs. Eleven patients (7 MoMHR and 4 THA) did not complete the MRI protocol for liver imaging due to MRI scanner intolerance. Circulating hypothalamo-pituitary axis and downstream hormones and pituitary volume were similar between groups (Table 3, P>0.05). Fasting serum glucose, insulin and glucose to insulin ratio were also similar between groups (P>0.05).

## Discussion

We conducted an in-depth cross-sectional health screen to identify those organ systems that may be susceptible to chronic metal exposure at levels representative of patients with well-functioning MoMHR devices to inform clinical monitoring guidelines and the design of larger scale screening studies now required of prosthesis manufacturers. Our data suggest mixed health effects, including potentially positive effects on systemic bone mass, but potentially deleterious effects on left ventricular function. Our data suggest that the cross-sectional study designs required of manufacturers by the FDA, and the clinical follow-up of asymptomatic patients with elevated metal levels, should prioritize these organ systems. In contrast, we found no evidence to implicate neuropsychological, psychological, renal, or endocrine dysfunction at this level of metal exposure. We also found no evidence of association between metal exposure and hepatic dysfunction, although we did find MRI signal in the liver and spleen consistent with metal deposition. Our data also suggest that the FDA-required studies should include data collection on concurrent medication that may modulate the urinary fractional excretion of chromium or cobalt.

This study was conducted in a clinically healthy population and the patients underwent a high level of investigational phenotyping. The MoMHR patients studied showed good external validity to the general MoMHR population as they reflected the younger arthroplasty patient, and the majority were male. The study also reflected well-functioning devices, as the majority of prostheses were of the Birmingham design, most subjects had an Oxford hip score greater than 42 that defines an excellent outcome, and they had a blood metal concentration that was below the MHRA 7 µg/L threshold. Levels of both cobalt and chromium in blood in normal health are typically ≤0.30 µ/L (4 nmol/L) [18]. Blood metal concentrations rise after MoMHR insertion, and usually peak during the first year after surgery at 1.3 to 2.5 µ/L [18], [19]. In the long term after surgery metal levels remain elevated above physiological levels in well-functioning implants, with mean levels at between 0.8 and 2.5 µg/L in well-functioning implants up to 10 years after surgery [18], [20], [21]. Local tissue levels at the site of the prosthesis are higher. Kwon et al reported cobalt and chromium levels within the hip joint synovial fluid of patient with well-functioning implants of 1 to 158 µg/L and 3 to 230 µg/L, respectively [22]. Cobalt and chromium levels in the synovial fluid of patients with failing prostheses may reach 24,000 µg/L and 263,000 µg/L, respectively,_ENREF_10 and blood metal can reach several hundred or thousands of µg/L [23], [24], [25].

These clinical findings confirm clinically our previous observations *in-vitro* that cobalt and chromium concentrations within the relevant systemic range after MoMHR may affect systemic bone cell function [13]. One explanation for this observation is that chronic metal exposure after MoMHR has a direct systemic anti-resorptive effect on bone through suppression of osteoclast number or activity, resulting in increased secondary mineralization of bone in a similar manner to that seen with bisphosphonate therapy [26]. This mechanism is supported by the lower TRAP-5b levels in the MOMHR patients, suggesting reduced osteoclast number. The effect might also be exerted through an indirect mechanism, as chromium increases insulin sensitivity [27], modulating the anabolic skeletal effects of both insulin and parathyroid hormone [28], [29]. However, our glucose and insulin assay data suggests that there was no overt difference in insulin sensitivity between the patient groups. An alternate explanation for our finding is that metal deposition within bone artifactually raises the measured x-ray attenuation. This effect is seen after strontium ranelate therapy because the atomic weight of strontium that is twice that of calcium [30]. However, both cobalt and chromium have atomic weights that are similar to calcium, and thus are unlikely to have a significant impact on x-ray attenuation or BMD measurements in this study.

An association between cobalt exposure and cardiomyopathy was first identified following its addition to beer as a foam-stabilizing agent in Quebec in the 1960’s [31]. Linna et al have previously shown that cobalt-workers had echocardiographic evidence of altered left ventricular relaxation and early filling versus unexposed controls [14]. The cobalt exposed workers in this study had mean blood cobalt levels of 2.5 µg/L and a mean duration of exposure of 8 years, that are similar to the exposure in the MoMHR population studied here. Our findings of differences in cardiac function are consistent with previous studies showing an association between accidental or occupational cobalt exposure and cardiomyopathy, although the differences in function we found between patient groups was subtle. There were no patients in either group with overt clinical cardiomyopathy by NYHA class, and only 1 patient in the MOMHR group had an LVEDD that exceeded the 56 mm upper limit of our normal reference range. Similarly, the lower limit of our EF% reference range (<50%) was exceeded by 2 patients in each study group.

The kidney is the primary route of excretion for metals, and thus is a potential site for toxicity as metal is concentrated at this site. Acute tubular necrosis, renal failure and chronic interstitial nephritis are reported following accidental ingestion of chromic acid [32]. In this study we found no evidence of renal injury using sensitive markers of renal tubular damage applied for the first time to the setting of MoMHR. Our finding of a clear difference in fractional excretion of cobalt versus chromium, and a potential interaction with diuretic therapy, requires further investigation and may have relevance for potential long-term relative accumulation of each metal. Cobalt inhibits tyrosine iodinase and prolonged exposure can result in thyroid hyperplasia and hypothyroidism [33]. Prolonged excess ingestion of chromium results in its deposition in the anterior pituitary and endocrine dysfunction, and increases insulin sensitivity [34]. The results of our studies suggest that at exposure concentrations found after successful MoMHR is not associated with endocrine dysfunction of the hypothalamo-pituitary axis hormones, including thyroid function, or alteration in pancreatic islet function.

The limitations of this study include potential pre-arthroplasty systematic differences between the populations associated with lifestyle choices, and potential differences in post-operative function associated with each device. In mitigation of the former, we found no evidence of systematic bias between groups to suggest differences in physical function that might suggest a pre-operative difference in BMD. Both groups had similar body mass index, lean mass, percentage fat mass, resting heart rates, and physical function by Oxford hip score. The groups also had similar pre-morbid 10-year fracture probabilities by FRAX, indicating similar risk factors for osteoporosis. Whilst unidentified differences in activity levels or other aspects of general health could be the cause of the difference in BMD, rather than effect of metal exposure, this does not adequately explain the associated suppression of bone turnover markers, and would be inconsistent with the observed poorer cardiac function in the MoMHR group. In relation to the second limitation, recent randomized clinical trial data indicates that hip resurfacing does not confer better physical functional or higher activity levels versus conventional arthroplasty in patients with arthritis [35], [36].

In conclusion, our data indicates that hip resurfacing associates with subtle structural or functional differences in multiple solid organs at 8 years after surgery in asymptomatic patients with well-functioning MoMHR versus those who received conventional THA. Our data suggest that long-term epidemiological studies in large populations, such as those available through joint registries, are required to quantitate the risk of clinical disease, and that these studies should include cardiac and musculoskeletal endpoints. Finally, our data suggest that the studies required of manufacturers by the FDA should be conducted over an extended period if the late clinical consequences of metal exposure are to be accurately quantitated.
